# Predictors of participants’ retention—socioeconomic factors or nonadherence: insights from a urological clinical prospective study

**DOI:** 10.1186/s13063-022-06901-w

**Published:** 2022-12-02

**Authors:** Allison J. Wheeler, Harshit Garg, Dharam Kaushik, Ahmed Mansour, Deepak Pruthi, Michael A. Liss

**Affiliations:** Department of Urology, University of Texas Health San Antonio, 7703 Floyd Curl Drive, San Antonio, TX 78229 USA

**Keywords:** Clinical trials, Socioeconomic factors, Humans, Subject retention

## Abstract

**Background:**

To investigate various patient-level variables, specifically socioeconomic status, as risk factors for withdrawal in a recently completed clinical study. We specifically investigated a non-interventional prospective study assessing the role of novel imaging as a biomarker for cancer upgradation in prostate cancer for this objective.

**Methods:**

In this retrospective analysis, we assessed the association between various patient-level factors including clinic-demographic factors, socioeconomic status, and the number of non-adherences with the participants’ retention or withdrawal from the study. For socioeconomic status (SES), we used the zip code–based Economic Innovation Group Distressed Community Index (DCI) which classifies into five even distress tiers: prosperous, comfortable, mid-tier, at-risk, or distressed. Low SES was defined as those with a DCI Distress tier of at-risk or distressed. We compared values between the two retention and withdrawal groups using *t*-test, chi-square test, and logistic regression analysis.

**Results:**

Of 273 men screened, 123 men were enrolled. Among them, 86.2% (106/123) retained through the study whereas 13.8% (17/123) withdrew from the study. The mean (SD) age was 64 (6.4) years. Overall, 31.7% (39/123) were Hispanics and 24.3% (30/123) were African Americans. The median (IQR) DCI score was 34 (10.3, 68.1) and 30.8% (38/123) of patients belonged to low SES. The median DCI score in participants who retained in the study was statistically similar to those who withdrew from the study (*p*=0.4). Neither the DCI tiers (*p*=0.7) nor the low SES (*p*=0.9) were associated with participants’ retention or withdrawal of the study. In terms of non-adherence, all participants in the withdrawn group had at least one non-adherent event compared to 48.1% in the retained group (*p*<0.001). Repetitive non-adherence was significantly higher in participants who withdrew from the study vs those who retained in the study [88.2% vs 16.9%, *p* <0.001]. On multivariate logistic regression analysis, the number of non-adherences (OR=12.5, *p*<0.001) and not DCI (OR=0.99, *p*=0.7) appeared to be an independent predictor for participants’ retention or withdrawal from the study.

**Conclusions:**

Expanding diverse inclusion and limiting withdrawal with real-time non-adherence monitoring will lead to more efficient clinical research and greater generalizability of results.

**Supplementary Information:**

The online version contains supplementary material available at 10.1186/s13063-022-06901-w.

## Background

Clinical research studies lead to discovery and improvements in the prevention, diagnosis, and treatment of disease [1]. As of December 10, 2021, ClinicalTrials.gov lists 397,984 studies across a total of 220 countries, with 78% being interventional trials and 22% being observational human research studies [2]. Unfortunately, marginalized groups are underrepresented in clinical research even though they face a greater disease burden [3, 4]. Traditional enrollment strategies target affluent patients thought to be more engaged and less likely to withdraw from research studies [4]. Frequently, research teams have emphasized strategies to overcome enrollment barriers, while we focus on the substantial issue regarding participants’ retainment and study completion [5–7].

Two of the most critical components for completing clinical research are the retention and protocol adherence of participants. Unfortunately, retaining subjects and ensuring adherence to the protocol are major challenges for clinical researchers [8–10]. The retention and adherence of subjects is necessary for the credibility, accuracy, and validity of research studies as low retention and adherence can reduce statistical power, lead to selection bias, and influence the significance of a study’s findings [8, 9, 11, 12] Additionally, a high number of subjects who withdrew and non-adherences can lead to a waste of resources and funds, create an excess burden on research coordinators, and ultimately lengthen the duration of studies causing delays in advancing medical knowledge and providing better care to patients [11].

While subject retention and protocol adherence is essential for successful research studies, little is known about the specific factors that influence subjects to complete a study and adhere to the protocol. Few studies have suggested socioeconomic status as a factor for patient inclusion in the clinical trial. Factors such as low education level, traditional and cultural beliefs, access to health care, and issues pertaining to health insurance are often prevalent in patients with low socioeconomic status, and hence, this coupled with providers’ perception of their inability to complete the study protocol often excludes them to clinical trials [4, 5]. Though various studies have proposed strategies to address socio-economic barriers in clinical trials [6], another major factor associated with participant retention in the study is repetitive non-adherence. Communication strategies and monetary incentives have been proposed to improve participants’ retention and decrease non-adherence to study protocol in a few studies [8]. Hence, it becomes imperative to evaluate the impact of socio-economic factors on participants’ retention and completion of the study protocol, so that these could be understood and addressed effectively. We investigate various patient-level variables as risk factors for withdrawal in a recently completed non-interventional study on prostate imaging in men with prostate cancer to specifically explore socioeconomic factors as potential barriers.

## Material and methods

### Population and setting

**Fig. 1:**
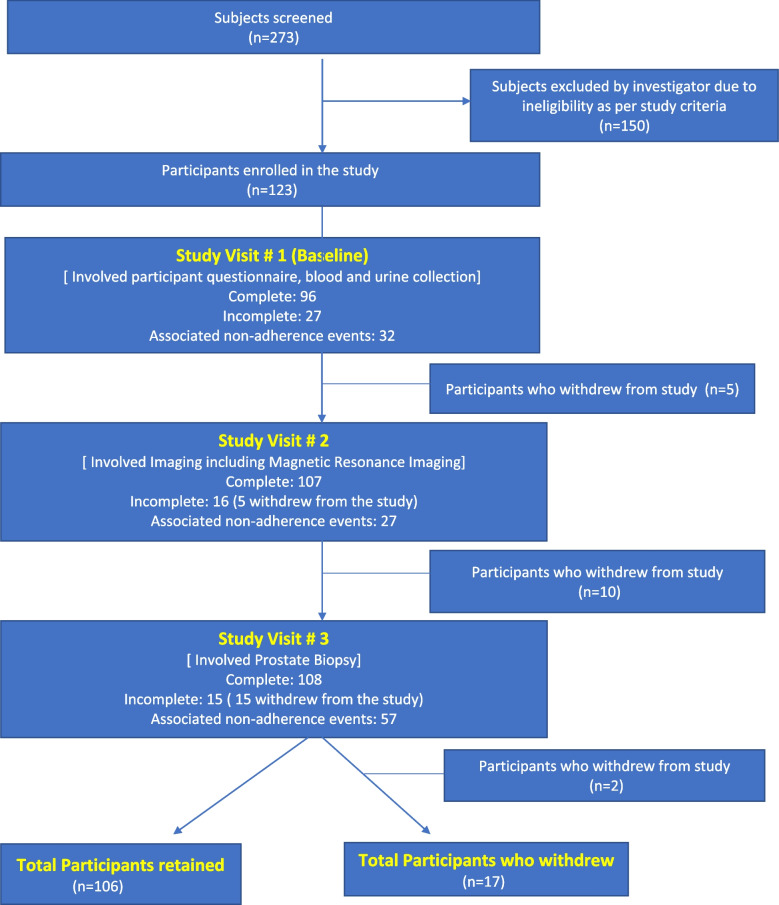
Cohort flow chart

We enrolled men in a prostate cancer study exploring novel imaging to predict cancer outcomes from January 2016 to June 2019 under IRB HCS20150160H. The study was done at a tertiary care university hospital in the USA. An IRB exempt determination was obtained, HSC20200572E, to perform a retrospective chart review study to investigate risk factors for withdrawal of the participants. Figure 1 depicts the study flowchart. The prostate imaging study enrolled 123 prostate cancer patients on active surveillance. The study aimed to understand the role of novel magnetic resonance imaging (MRI) protocol in the prediction of cancer upgradation. The study involved primarily 3 visits—a baseline visit for blood and urine collection and questionnaire completion, a second visit which was the research visit for MRI wherein research imaging MRI was acquired at the same time as the standard of care MRI (multiparametric-MRI), and the third visit for the baseline prostate biopsy for cancer diagnosis. All these visits were scheduled during the working day, and one of these three visits was the visit for research purposes specifically. The participants were required to travel to the study site. Once enrolled, the patients were followed up as per the protocol of active surveillance in prostate cancer, and the outcome of cancer upgradation was determined. The duration of the study was 3 months.

### Primary outcome

For each, we assessed whether they retained [defined as the proportion of patients that remained in the study to completion] on the study or withdrew [defined as the proportion of patients that did not complete the study] and the reason for withdrawal if applicable.

### Primary predictor

Our primary predictor of interest was low socioeconomic status (SES). For each subject, their zip code was entered into the Economic Innovation Group Distressed Community Index (DCI) which ranks communities across seven measures: no high school diploma, housing vacancy rate, adults not working, poverty rate, median income ratio, change in employment, and change in establishments [13]. (Supplementary Table 1) These rankings are “averaged and weighted equally” and “then normalized into a final score that ranges from approaching 0 (most prosperous) to 100 (most distressed).” Zip code areas are further classified into five even distress tiers: prosperous, comfortable, mid-tier, at-risk, or distressed [13]. We further defined low SES as those with a DCI distress tier of at-risk or distressed.

### Secondary predictors

In addition, we collected data on various clinic-demographic variables including age, race, ethnicity, body mass index (BMI), smoking history, comorbidities using the Charlson Comorbidity Index (CCI), and time from prostate cancer diagnosis to enrollment in the prostate cancer imaging study. For each participant, regardless of retention outcome, we identified instances of non-adherence, defined as any incomplete study procedure or issues with scheduling visits. The reason for each non-adherence was documented.

### Statistical analysis

Continuous variables are presented as the median (interquartile range) or mean (standard deviation) as appropriate. Categorical variables as proportions. Categorical variables were compared using the chi-square test (or Fisher’s exact test for lower frequencies) and continuous variables were compared using Students’ *t*-test or Mann-Whitney test as appropriate. The correlation between the two variables was assessed using Spearman’s rank correlation or Pearson’s coefficient as appropriate. We used logistic regression analysis to identify independent predictors associated with participants’ retention or withdrawal from the study. All tests were two-sided and *p*≤0.05 was considered statistically significant. The statistical analysis was performed using IBM SPSS Statistics software (version 25.0, Chicago, IL).

## Results

### Demographics

We screened 273 men and enrolled 123 participants for a participation rate of 45%. Among these 123 participants, 86.2% (106/123) retained through the study and completed the study protocol, whereas 13.8% (17/123) withdrew from the study (Fig. 1). Supplementary Table 2 provides details on the reasons for the participants’ withdrawal and most participants were withdrawn from the study by the investigator in view of poor compliance with the study protocol.

The mean (SD) age of the study cohort was 64 (6.4) years, and the mean CCI was 4.2 (0.9). Among the participants, 31.7% (39/123) were Hispanics and 24.3% (30/123) were African Americans.

### Socioeconomic status and study retention

The median (IQR) DCI score of the study cohort was 34 (10.3, 68.1) with 39% (48/123) participants falling in the prosperous tier, 17.1% (21/123) in the comfortable tier, 13% (16/123) in the mid-tier, 16.3% (20/123) in the at-risk tier, and 14.6% (18/123) in the distressed tier. Overall, 30.8% (38/123) of patients belonged to low SES.

**Fig. 2:**
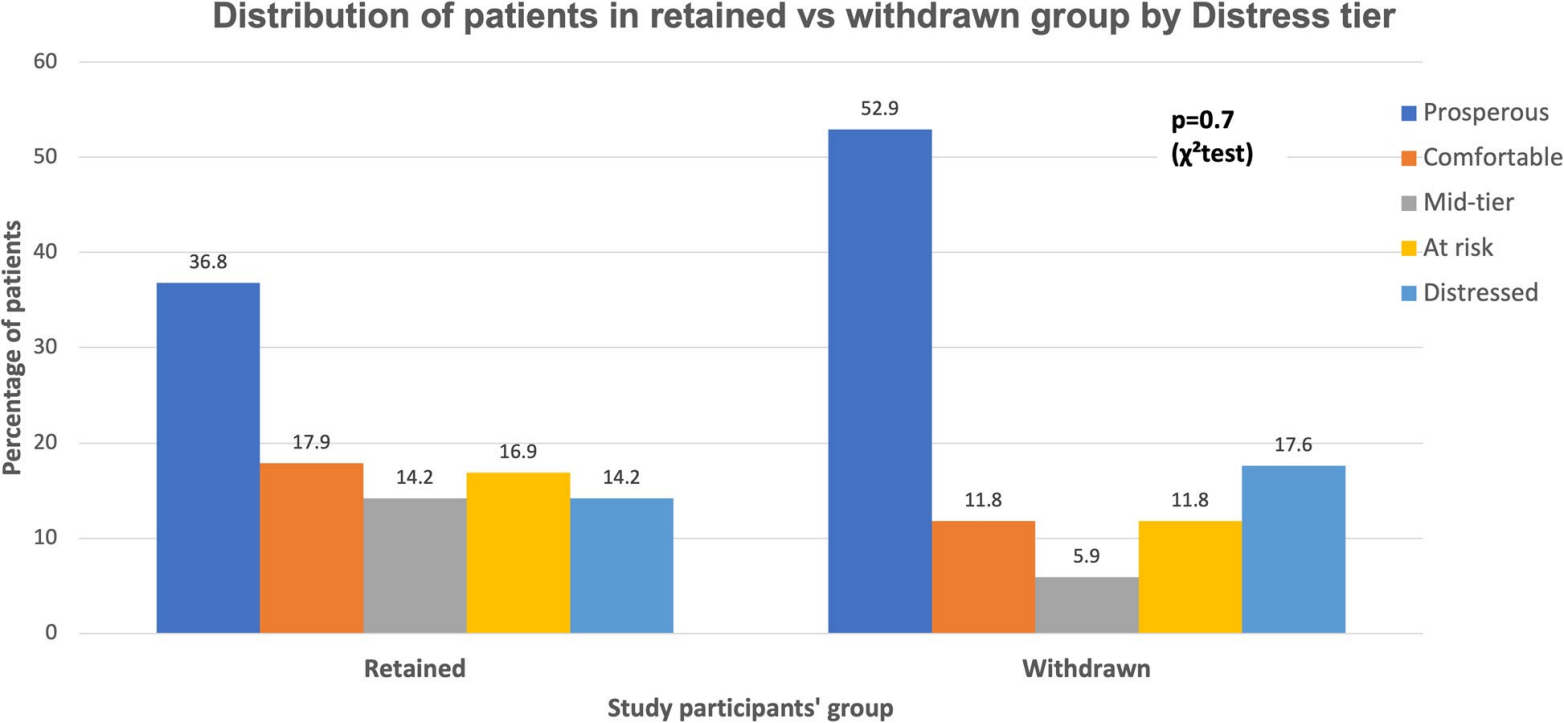
Distribution of patients in retained or withdrawn group by distress tier. We display a bar graph noting the proportion of patients that remained in the study to completion (retained) compared to those subjects who did not complete the study (withdrawn)

The median DCI score in participants who retained in the study was statistically similar to those who withdrew from the study [35.4 (10.3, 67.3) vs 17.1 (3.8, 68.1), *p*=0.4] (Table 1). The distribution of DCI scores was skewed to the left (Supplemental Fig. 1), but we did have a substantial distribution of DCI scores. For one participant, we utilized the county-level DCI score rather than the zip code–level DCI score due to this zip code not meeting the population, employment, and/or establishment data threshold for the DCI. We then grouped the DCI into distress tiers and studied the DCI tiers in relation to participants’ retention vs withdrawal from the study (Fig. 2). Neither the DCI tiers (*p*=0.7) nor the low SES (*p*=0.9) were associated with participants’ retention or withdrawal of the study (Table 1).

**Table 1:** Patient demographics and socioeconomic status characteristics in relation to study participants’ retention vs withdrawal

Demographics	Retained ***N***=106	Withdrawal ***N***=17	***p*** value
**Age**, *mean (SD), years*	64 (6.5)	63 (5.9)	0.8
**Ethnicity,** *n(%)*			0.8
Hispanic	34 (32.1%)	5 (29.4%)	-
Non-Hispanic	72 (67.9%)	12 (70.6%)	-
**Race,** *n(%)*			0.4
White	77 (72.6%)	15 (88.2%)	-
African American	28 (26.5%)	2 (11.8%)	-
Asian American or Pacific Islander	1 (0.9%)	0 (0.0%)	-
**BMI**, mean (SD), *kg/m*^*2*^	29.1 (4.4)	30.7 (3.5)	0.1
**CCI,** *mean (SD)*	4.2 (0.9)	4.1 (1.0)	0.6
**Smoking status,** ***n(%)***			0.8
Former smoker	38 (64.2%)	7 (41.2%)	-
Current smoker	18 (9.5%)	2 (11.8%)	-
Never smoked	49 (46.3%)	8 (47.0%)	-
**Days from diagnosis to enrollment,** *median (IQR)*	707 (384, 1340)	680 (393, 1045)	0.9
**Non-Adherence,** ***n(%)***			<0.001*
No	55 (51.9%)	0 (0%)	***-***
Yes	51 (48.1%)	17 (100.0%)	***-***
**Number of non-Adherences,** ***n(%)***			<0.001*
0	55 (51.9%)	0 (0%)	
1	33 (31.2%)	2 (11.8%)	
2	17 (16.0%)	8 (47.1%)	
3	1 (0.9%)	4 (23.5%)	
4	0 (0%)	3 (17.6%)	
**Socioeconomic status**			
**DCI score,** *median (IQR)*	35.4 (10.3, 67.3)	17.1 (3.8, 68.1)	0.4
**Distress tier,** ***n(%)***			0.7
Prosperous	39 (36.8%)	9 (52.9%)	
Comfortable	19 (17.9%)	2 (11.8%)	
Mid-tier	15 (14.2%)	1 (5.9%)	
At-risk	18 (16.9%)	2 (11.8%)	
Distressed	15 (14.2%)	3 (17.6%)	
**Low SES,** ***n(%)***			0.9
No	73 (68.9%)	12 (70.6%)	
Yes	33 (31.3%%)	5 (29.4%)	

*BMI* body mass index, *CCI* Charlson Comorbidity Index, *DCI* Distressed Communities Index

*p<0.05 is considered statistically significant

### Secondary indicators of clinical study retention

**Fig. 3:**
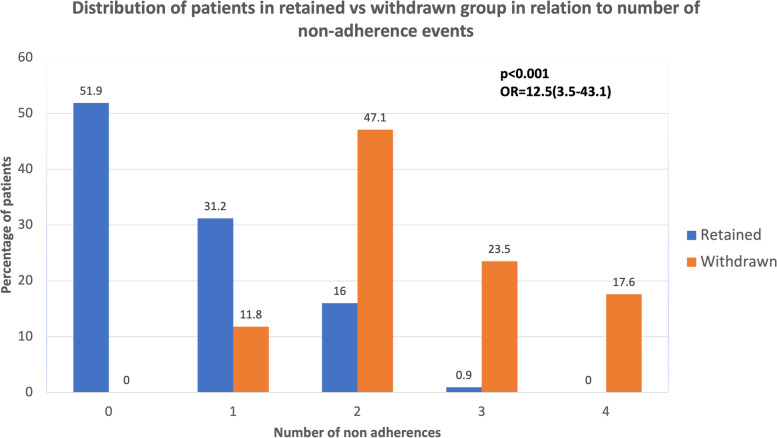
Distribution of patients in retained vs withdrawn group in relation to the number of non-adherent events. We present a bar graph to demonstrate the number and proportions of subjects that remain in the study compared to those who withdrew from the study represented by the number of protocol non-adherent events. We note that after 1 non-adherent event the proportion of subjects that withdraw markedly increases indicated a teachable moment after the first non-adherence

We further investigated clinico-demographic factors such as age, race, ethnicity, smoking status, BMI, CCI, and time from prostate cancer diagnosis to enrollment, and did not find any significant differences between the two groups (Table 1). However, the presence of a non-adherence, as expected, was significantly different between the two groups (*p*<0.001). Overall, 55% (68/123) had at least one instance of non-adherence. Of the participants with a non-adherence, 51% (35/123) participants had one non-adherence, 37% (25/123) participants had two, 7% (5/123) participants had 3, and (3/123) participants had four episodes of non-adherence. All participants in the withdrawn group had at least one non-adherent event compared to 48.1% in the retained group (*p*<0.001). Repetitive non-adherence, defined as more than one non-adherence, was significantly higher in participants who withdrew from the study vs those who retained in the study [88.2% (15/17) vs 16.9% (18/106),*p* <0.001]. The number of non-adherent events was also highly significant (range 0–4, *p*<0.001) (Fig. 3).

### Multivariate analysis

We performed a logistic regression analysis to identify independent predictors for participants’ retention vs withdrawal (Table 2). Age (*p*=0.4), race (*p*=0.3), ethnicity (*p*=0.3), BMI (*p*=0.6), CCI (*p*=0.5), smoking status (*p*=0.8), and days from prostate cancer diagnosis to enrollment (*p*=0.8) did not appear to be independent predictors. The SES studied using the DCI score also did not appear to be an independent factor for participants’ retention vs withdrawal (OR=0.99, *p*=0.7). Furthermore, the number of non-adherences appeared to be an independent predictor for participants’ withdrawal from the study [OR (95% CI) =12.5 (3.5, 40.5), *p*<0.001).

**Table 2:** Predictive factors for study participants’ withdrawal from the study using multivariate logistic regression analysis

Variable	Multivariate analysis	
	OR (95% CI)	***P*** value
Age	1.1 (0.9–1.4)	0.441
Race	2.6 (0.4–19.3)	0.343
Ethnicity	0.6(0.2–1.9)	0.383
Smoking	1.3 (0.7–26.2)	0.844
Body mass index	1.1 (0.8–1.3)	0.596
Charlson Comorbidity Index	0.7 (0.2–2.5)	0.543
Days from diagnosis to study enrollment	1.0 (0.9–1.1)	0.767
DCI score	0.99(0.96–1.02)	0.682
Number of non-adherence events	12.5(3.5–43.1)	<0.001*

*p<0.05 is considered statistically significant

*DCI* Distressed Communities Index

## Discussion

Our retrospective investigation of 123 men enrolled in a prostate cancer imaging study found that repeat non-adherence contributed to participant withdrawal more than SES. This finding is significant because it relates to the contemporary discussion regarding increasing diversity in clinical research.

Literature has consistently shown that clinical trials lack the inclusion of marginalized groups [4, 14, 15]. While the reasons for this are multifaceted and may be attributable to a lack of access, financial and logistical barriers, and general distrust, a significant reason may be due to provider perception. Fisher et al. suggest that “physician bias, false perceptions, and prejudices” have a significant impact on who they believe will comply with the often-demanding regimens involved with clinical research [16]. Our findings show that low SES, and/or minority patients, were not at higher risk for withdrawal and should not be excluded by physicians for this reason.

In our study, we used DCI as a measure for socio-economic evaluation. Various proposed measures for SES include insurance status, race, household income or employment status, or combination of these, however, the number of data points’ chosen to assess patients’ SES limit the ability to study the true impact of SES across various studies. A comprehensive evaluation of social, economic, and financial status is necessary to estimate SES and study its impact on health disparities and clinical trials. The Economic Innovation Group, a “bipartisan public policy organization”, introduced DCI, to understand the spatial distribution of US economic well-being. DCI combines seven distinct and complementary socio-economic indicators into a single score and creates a composite ranking by zip code. This score has been demonstrated to be an accurate measure of a community’s socio-economic distress and has been found to improve risk-adjusted outcomes after surgery [17].

One interesting but the anticipated finding is that participants with more than 1 non-adherent event were at a higher risk for eventually withdrawing from the research study. The majority of participants with two non-adherences withdrew from the study, nearly all participants with three non-adherences withdrew from the study, and all participants with four non-adherences withdrew from the study. It is important to note that not all non-adherences or withdrawals were directly attributable to the participant. Coordinator phlebotomy skill, inadequate screening, and clinic scheduling processes attributed to several non-adherences or withdrawals (Supplementary Table 2). Another important aspect regarding repetitive non-adherence is the investigators’ and participants’ perceptions regarding these events. As the number of non-adherence increases, while the participant might opt out of the study fearing their futile role in the study, the investigator might also withdraw these participants from the study, owing to undue burden on the research staff and introduction of bias in the study [18]. Non-adherence is known to increase variance, lower study power, and reduce the magnitude of treatment effects in a study and hence, International Society for CNS Clinical Trial Methodology (ISCTM) Working Group on Nonadherence in Clinical Trials proposed several recommendations to identify and mitigate its negative effects [18]. These measures predominantly include statistical analyses of nonadherence data and modification in study designs so as to address non-adherence at each step of the study.

We anticipate that future applications of our important finding would encourage real-time monitoring of non-adherent instances within clinical research studies in order to intervene early and prevent withdrawal from the study. The concept of real-time monitoring stems from successful remote monitoring to prevent and document adverse events, especially in phase 1, 2, and 3 clinical trials [19–22]. We specifically identified that the “adherence audit” should occur after the first non-adherence to identify the barriers of the participant or the coordinating team to implement immediate solutions. At that point, the team could assess study-team processes, environment, resources, or patient factors leading to the non-adherence and apply adaptive countermeasures to prevent further issues [12]. While our results on SES are favorable to expand enrollment, the finding by no means assumes there are no barriers caused by SES in clinical trials. We agree with acknowledging and addressing the barriers up front, to prevent non-adherence in a patient-centric manner [23]. Dockendorf et al advocated the use of digital health technologies comprising of smart dosing, outpatient sampling, and digital monitoring to increase participant retention with reduced burden on patients [23]. However, further research will be required to identify common barriers and solutions encountered in real-time during clinical trial activity.

A major limitation of this study is that our analysis derives from an observational study and not a clinical trial where intervention and randomization might further affect subject recruitment and retention. Furthermore, while we do have diversity of race and ethnicity, we lack age and gender diversity. Additionally, non-adherence data was gathered retrospectively resulting in unknown reasons for incomplete study procedures, a lack of details regarding the reason and time-point of withdrawal and therefore the decision to count any incomplete study procedure as a non-adherence regardless of retention status, and missing information regarding the barriers faced by subjects and coordinators. As the participants were required to travel to the study site, their travel distance could also be a potential factor for non-adherence to the study, however, this could not be studied. Also, small sample size and low number of events (withdrawals) need larger studies for definitive inference. Future studies are needed to examine various populations and different types of clinical research studies, specifically clinical trials that include real-time non-adherence data collection.

## Conclusion

Our findings show that low SES subjects are not at greater risk for withdrawal and researchers should actively make efforts to expand their participation in research. Our findings also highlight the importance of early intervention following a subject’s first non-adherence from the protocol to prevent withdrawal. Expanding diverse inclusion and limiting withdrawal with real-time non-adherence monitoring will lead to more efficient clinical research and greater generalizability of results.

## Supplementary Information

**Additional file 1: Supplementary Table 1.** Components of the Distressed Communities Index. **Supplementary Table 2.** Description of non-adherent events and withdrawals. **Supplementary Figure 1.** *Distribution of DCI scores*. We display a histogram to describe the overall distribution of distressed community index (DCI) scores in our population.

## Data Availability

The datasets used and analysed during the current study are available from the corresponding author on reasonable request

## Abbreviations

BMI: Body mass index
DCI: Distressed Community Index
IRB: Insitute Review Board
MRI: Magnetic resonance imaging
SES: Socioeconomic status
