# Supplementary material for: Predictors of participants’ retention—socioeconomic factors or nonadherence: insights from a urological clinical prospective study
Source: Trials. 2022 Dec 2;23:970. doi: 10.1186/s13063-022-06901-w (PMC9716754; doi:10.1186/s13063-022-06901-w)

**Supplementary Material**

**Supplementary Table 1: Components of the Distressed Communities Index^13^**

| **Component** | **Definition** |
| --- | --- |
| No high school degree | Percent of population age ≥ 25 years without a high school diploma |
| Housing vacancy rate | Percent of habitable housing that is unoccupied and not for seasonal, recreational, or occasional use |
| Adults not working | Percent of the population age ≥ 16 years not currently in work |
| Poverty rate | Percent of the population living under the poverty line |
| Median income ratio | Ratio of a geography’s median income to that of its state |
| Change in employment | Percent change in the number of jobs (2010–2013) |
| Change in business establishments | Percent change in the number of business establishments (2010–2013) |

**Supplementary Table 2:** Description of non-adherent events and withdrawals

| **Non-Adherent Event** | **Reason** |
| --- | --- |
| *Baseline Procedures* |  |
| Incomplete questionnaires (4) | Subject inadvertently did not complete some or all questionnaires (3) |
|  | Unknown reason (1) |
| Incomplete blood collection (21) | Subject refused blood draw (6) |
|  | Coordinator attempted blood draw, but was unsuccessful (4) |
|  | Incomplete blood collection due to unsuccessful draw (8) |
|  | Not attempted (1) |
|  | Unknown reason (2) |
| Incomplete urine collection (7) | Subject refused urine collection (1) |
|  | Subject was unable to provide urine (2) |
|  | Not attempted (1) |
|  | Urine was collected but discarded due to transportation issue (1) |
|  | Unknown (2) |
| *Imaging (MRI)* |  |
| Incomplete MRI (16) | MRI attempted, but not tolerated by subject (2) |
|  | Subject refused MRI (3) |
|  | Subject withdrawn prior to MRI (5) |
|  | MRI was not attempted due to recommendation by MRI tech (3) |
|  | Only partial completion of MRI due to intolerability by subject (3) |
| Issues scheduling MRI (11) | Subject had to be rescheduled at least once (9) |
|  | Subject no showed at least once (1) |
|  | Subject required multiple contact attempts to schedule (1) |
| *Prostate Biopsy Procedure* |  |
| Incomplete prostate biopsy (15) | Subject refused biopsy (5) |
|  | Subject withdrawn prior to biopsy (10) |
| Issues scheduling prostate biopsy (42) | Subject had to be rescheduled at least once (37) |
|  | Subject no showed at least once (1) |
|  | Subject moved out of state during participation in study (1) |
|  | Subject had issues with insurance coverage (1) |
|  | Subject required multiple contact attempts to schedule (2) |
| **Withdrawals** |  |
| Following Baseline Procedures (5) | Physician decision due to non-adherence to protocol (5) |
| Following MRI (10) | Physician decision due to non-adherence to protocol (4) |
|  | Patient decision (2) |
|  | Ineligible, i.e. screen failure (2) |
|  | Lost to follow up, i.e. unable to contact (1) |
|  | Physician decision due to intercurrent illness (1) |
| Following Biopsy (2) | Physician decision due to non-adherence with protocol (1) |
|  | Ineligible, i.e. screen failure (1) |

**Supplementary Figure 1.** Distribution of DCI scores


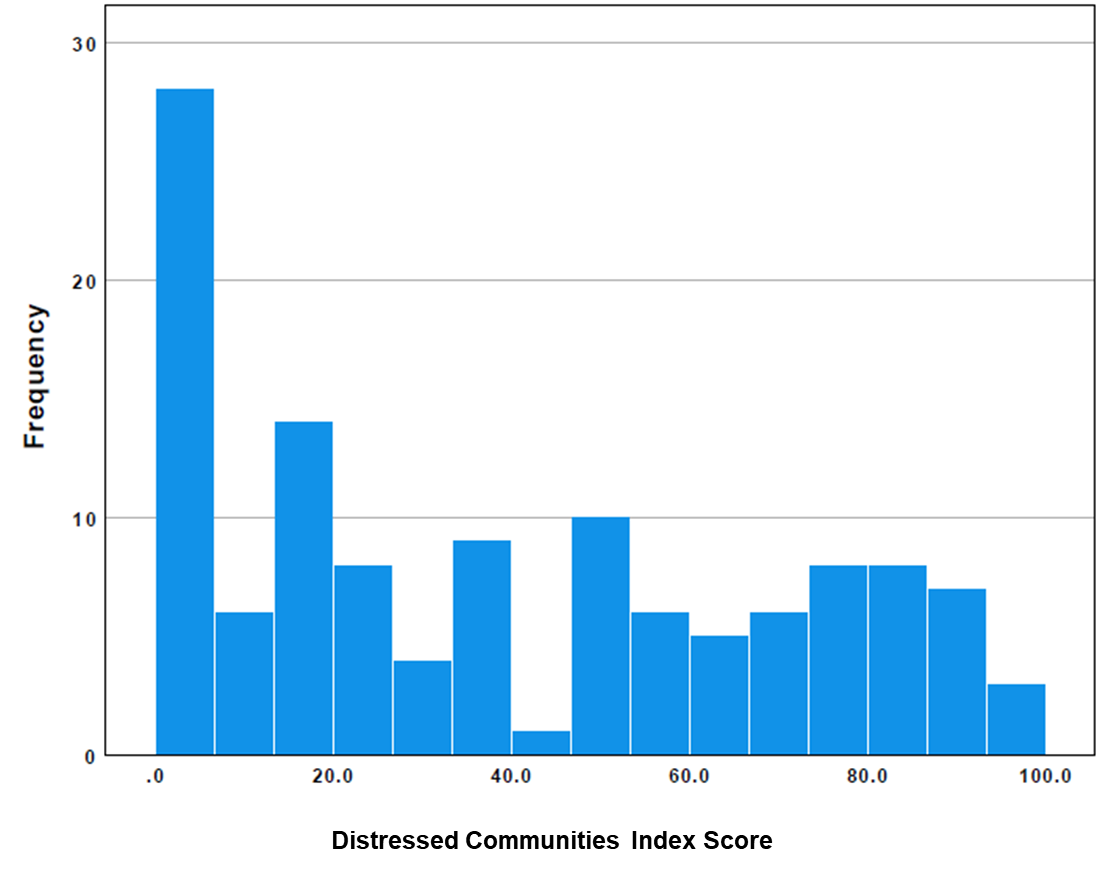

Supplement: Supplementary file 1 — Additional file 1: Supplementary Table 1. Components of the Distressed Communities Index. Supplementary Table 2. Description of non-adherent events and withdrawals. Supplementary Figure 1. Distribution of DCI scores. We display a histogram to describe the overall distribution of distressed community index (DCI) scores in our population. [file 13063_2022_6901_MOESM1_ESM.docx]
